# The Role of Bronchoscopic Therapy in Bronchial Granular Cell Myoblastoma: Presentation of Three Cases

**DOI:** 10.1155/DTE.2.223

**Published:** 1996

**Authors:** C. L. van Felius, P. E. Postmus, F. Beaumont, W. Strankinga, J. Teengs, T. G. Sutedja

**Affiliations:** 1 Department of Pulmonology Free University Hospital P.O. Box 7057 Amsterdam 1007 MB The Netherlands; 2 Department of Pulmonary Diseases Groot Ziekengasthuis Den Bosch The Netherlands; 3 Department of Pulmonary Diseases BovenlJ Hospital Amsterdam The Netherlands; 4 Department of Pulmonary Diseases Kennemer Gasthuis Haarlem The Netherlands

## Abstract

Tracheobronchial granular cell myoblastoma (GCM) is rare. Although the tumor has some malignant properties, it is considered benign, and there is no consensus regarding treatment. Three cases are reported here. Bronchoscopic treatment in patients with GCM may be attempted as a first approach in case the tumor is intraluminal.


*Diagnostic and Therapeutic Endoscopy*, 1996, Vol. 2, pp. 223–227
Reprints available directly from the publisher
Photocopying permitted by license only

© 1996 OPA (Overseas Publishers Association)
Amsterdam B. V. Published in The Netherlands
by Harwood Academic Publishers GmbH
Printed in Singapore

### The Role of Bronchoscopic Therapy in Bronchial Granular Cell Myoblastoma: Presentation of Three Cases

C. L. VAN FELIUS, P. E. POSTMUS, F. BEAUMONT, W. STRANKINGA, J. TEENGS, and T. G. SUTEDJA

*Department of Pulmonology, Free University Hospital, Amsterdam, (C.L.V.F., P.E.P., T.G.S.), Department of Pulmonary Diseases, Groot Ziekengasthuis, Den Bosch (F.B.), Department of Pulmonary Diseases, BovenIJ Hospital, Amsterdam (W.S.), and Department of Pulmonary Diseases, Kennemer Gasthuis, Haarlem (J.T.), The Netherlands*

*(Received August 3, 1995; in final form December 4, 1995)*

Tracheobronchial granular cell myoblastoma (GCM) is rare. Although the tumor has some malignant properties, it is considered benign, and there is no consensus regarding treatment. Three cases are reported here. Bronchoscopic treatment in patients with GCM may be attempted as a first approach in case the tumor is intraluminal.

**KEY WORDS:** Granular cell myoblastoma, carcinoid, bronchoscopic electrosurgery

### INTRODUCTION

The first report on granular cell myoblastoma (GCM) was by Abrikossoff in 1926 and heralded a considerable quantity of case reports, reviews, and histologic investigations. Frenckner, in 1938, described the first GCM in the respiratory tract. Since then, more than 60 cases have been reported (1). Multicentric tracheobronchial localization of GCM has also been described (2,3). GCM is characterized macroscopically by a relative small size, usually less than 5 cm in diameter, ill-defined margins, lack of encapsulation, firm texture, and yellowish or greyish color (4,5). The tumor appears to infiltrate into the surrounding tissue, causing a rather characteristic pseudo-epitheliomatous hyperplasia of the overlying epithelium. Microscopy shows closely packed nests of large cylindrical cells, with eosinophilic particles of various sizes and distribution. Currently it is believed that GCM originates from either a Schwann cell or its mesenchymal precursor (5). GCM is characterized by local growth, and there is no tendency for metastasizing to regional or mediastinal lymph nodes. So, from the oncologic point of view, there seems to be no need for a prompt surgical approach. Review of the literature on the treatment of this benign tumor showed that there is no consensus regarding treatment (1,4). Surgery has been advocated as the treatment of choice (6). Others favored local treatment of GCM (4). We present three patients with tracheobronchial localization of GCM and analyze the role of bronchoscopic treatment herein.

### CASE REPORTS

### Case 1

A 36-year-old Caucasian woman had a history of "bronchitis" during infancy. For several months she had been complaining of shortness of breath and cough, which persisted despite treatment with inhaled steroids and $\beta_2$-agonists. Bronchoscopy was performed and a 1.5 by 2 cm yellow tumor 4 cm above the carina in the membranous part of the trachea was found. Histologic examination revealed granular cell myoblastoma.

The patient was referred to our hospital for further treatment. Physical examination, laboratory results, and chest x-rays were normal. High-resolution computed tomography (HRCT) of the thorax showed nonspecific changes of the right and left lower lobe in the parenchymal setting. A 2-cm lesion with possible tumor extension dorsal in the trachea was seen on the magnetic resonance imaging (MRI) scan. Because of tumor size and location, partial

Address for correspondence: C.L. van Felius, M.D., Department of Pulmonology, Free University Hospital, P.O. Box 7057, 1007 MB Amsterdam, The Netherlands. Tel. 31-20-4444782; Fax: 31-20-4444328.

tracheal resection and tracheoplasty were performed and the anastomosis was covered with a pericardial patch. The margins of the resected specimen were not free from tumor microscopically. The postsurgical period was complicated by mediastinitis and anastomotic dehiscence. The pericardial patch loosened and had to be removed bronchoscopically because of airway obstruction. Despite microscopic residual disease, the patient is doing well without local recurrence for a follow-up period of 2 years. CT scan and bronchoscopy were repeatedly negative for recurrent disease.

### Case 2

A 64-year-old Caucasian woman had a history of chronic obstructive pulmonary disease and hypertension. She experienced progressive dyspnea, and she complained of coughing up whitish sputum and of left-sided pectoral chest discomfort. She had no fever. She had a 35 pack-year smoking history. The patient was treated with inhaled salbutamol and beclomethasone. Chest x-rays showed an infiltrate of the left upper lobe.

Bronchoscopy was performed and no abnormality was found on the left side, but a whitish tumor less than 1 $cm^2$ was found on the carina of the right upper lobe (Fig. 1A). Biopsy revealed GCM. HRCT did not show tumor extension peribronchially, and no enlarged lymph nodes were found. Bronchoscopic electrosurgery was performed using the argon beam coagulation technique under local anesthesia (Fig. 1B). Follow-up has been uneventful, and she is still free of disease after 1 year. Repeated bronchoscopic examination was negative for GCM (Fig. 1C).

### Case 3

A 40-year-old Caucasian woman had a history of partial gastrectomy because of leiomyoma, hysterectomy because of uterus myomatosus, and asthma treated with inhaled beclomethasone and salbutamol. She had been well until 3 months before admission when she complained of dyspnea and coughing up yellowish sputum. She denied chest pain, hemoptysis, and fever. There was a 2-kg weight loss. She had been smoking 20 cigarettes daily until 1 year ago.

On physical examination, there were increased breath sounds over the right upper lobe and a slightly prolonged expiration. Laboratory tests and chest x-rays were normal. Because of persistent cough, a bronchoscopy was performed.

Three abnormalities were found: a white lesion on the lateral wall of the tracheobronchial corner extending to the right upper lobe orifice, a reddish pedunculated tumor on the dorsal wall of the intermediate bronchus, and a 4-mm aspirated barley-corn in the bronchus intermedius. The latter was easily removed.

On histologic examination the tracheobronchial lesion was a GCM (Fig. 2A), and the lesion in the intermediate bronchus was a typical carcinoid. HRCT showed that both tumors were intraluminal, and no mediastinal lymph node enlargement was seen. Because of the superficial growth of both lesions, bronchoscopic treatment with electrosurgery was successful (GCM: Fig. 2B) in obtaining a complete response.

### DISCUSSION

GCM is a rather uncommon tumor predominantly found in the skin, subcutaneous tissue, oral mucosa, tongue, and breast, in which 8 to 16% were multifocal (7). Less frequent localizations in other organs have been reported in many case studies. Approximately 5 to 10% of patients with tracheobrochial GCM have multiple lesions (8). The age of most patients with a tracheobronchial GCM varies from 30 to 50 years, and there is an equal distribution between men and women. Patients usually complain of cough, dyspnea, chest pain, recurrent pulmonary infections, and sometimes hemoptysis (4). Radiographic investigation frequently discloses infiltrates or atelectasis due to tumor obstruction.

In a review of 13 cases of bronchoscopic removal of GCM, resection was advocated for tumors larger than 8 mm, and in patients with irreversible damage of the pulmonary parenchyma distal to the lesion (6). Bronchoscopic therapy was considered as a treatment alternative if tumor size was less than 8 mm, or if major surgery was contraindicated. Multicentric lesions less than 10 mm should be removed bronchoscopically, and those larger than 10 mm surgically (lobectomy, pneumonectomy, or bronchoplasty). HRCT may be useful in determining tumor extent in cases of intraluminal tumors (9,10) and in assessing the quality of pulmonary parenchyma distal to the lesion (11).

The finding of tumor extension is important as bronchoscopic treatment is only potentially curative in tumors not extending to the bronchial mucosal layers. This was particularly true in patients with superficial squamous cell lung cancer. Whether the same situation may apply to GCM is unknown (12,13).

Successful local therapy of GCM using the $CO_2$ laser, the Nd-YAG laser, and endobronchial curettage have been reported (4,8,14). However, a report of recurrent disease 5 months after $CO_2$ laser therapy of a 2 by 1 cm large tracheal lesion has also been published (1). The same article referred to two successful cases of $CO_2$ laser treatment. In

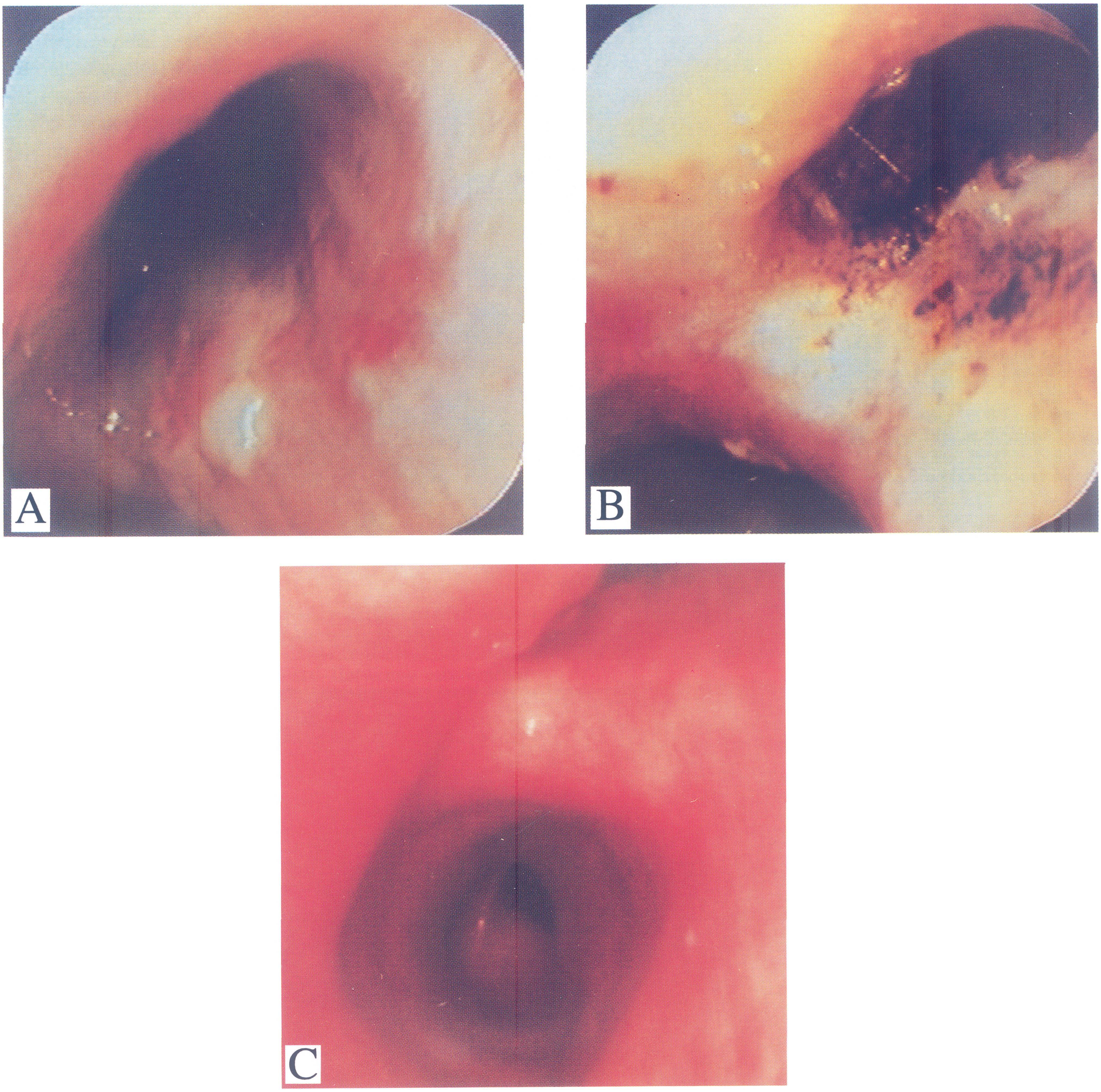

**Figure 1** A. A superficial whitish tumor (histologic analysis: GCM) of the right upper lobe carina. B. Immediately after treatment with argon gas coagulation. C. Bronchoscopic view of the same area 1 year after treatment. No residual GCM in the histologic specimens.

all our patients, the tumor proved to be strictly intraluminal, and the MRI findings for the first patient were false-positive. Tracheoplasty in our first patient was complicated by mediastinitis and despite microscopic residual disease, recurrence has not occurred. This seems to fit with GCM characteristics of a more benign nature in the tracheobronchial tree in contrast to rare malignant forms as have been described in other organs (15).

Our two remaining patients were treated successfully with electrosurgery as tumors were strictly intraluminal. The role of bronchoscopic treatment, e.g., electrosurgery may become increasingly important in tumors located intraluminally (16), as we have been expanding our data in treating occult squamous cell cancer and typical bronchial carcinoid. To our knowledge, the third patient is the first case of coexisting GCM and typical carcinoid of the

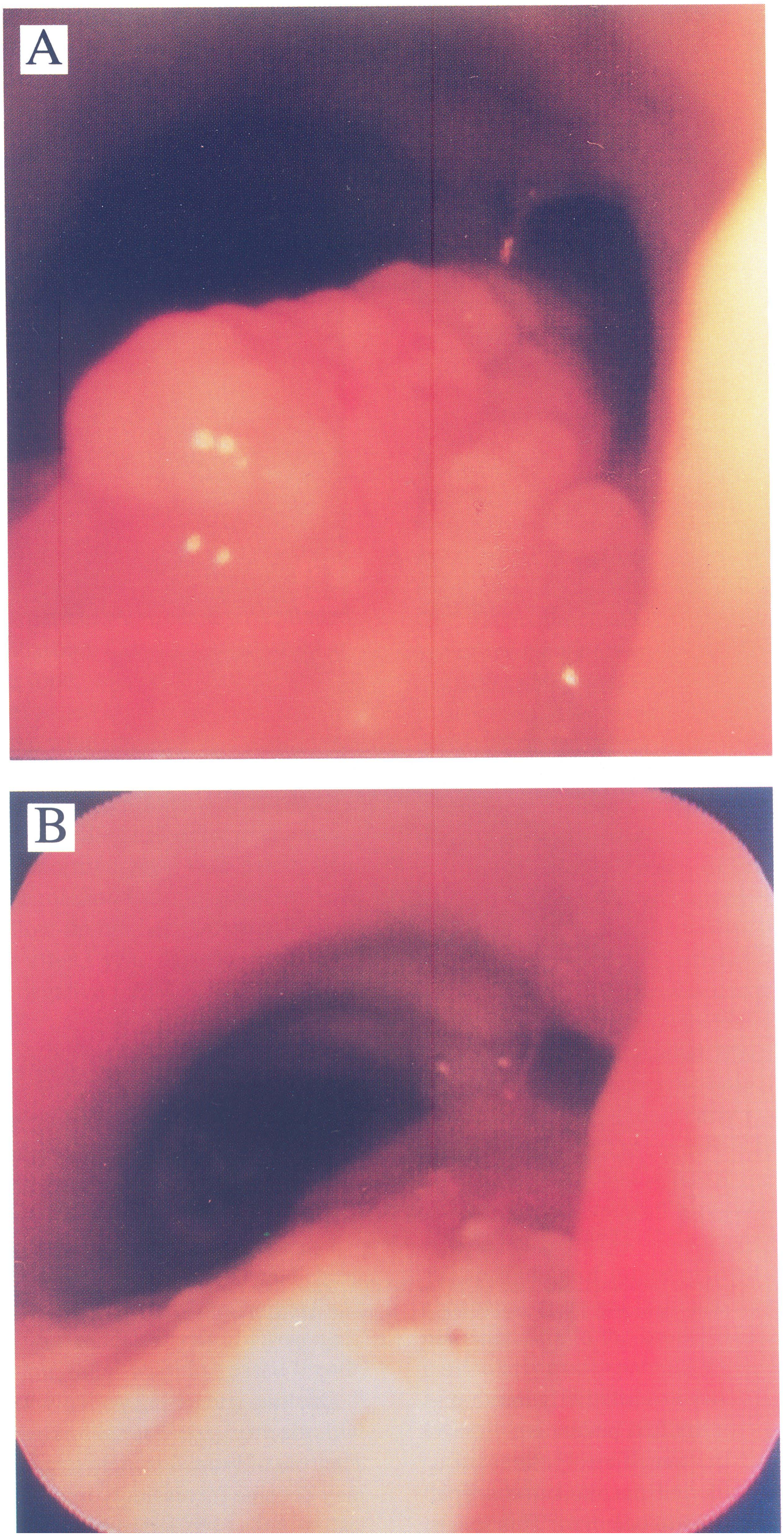

**Figure 2** A. A yellowish tumor (histologic analysis: GCM) on the dorsal wall of the right main bronchus extending to the right tracheobronchial corner. B. View 4 months after bronchoscopic electrosurgery. No residual GCM in the histologic specimens.

bronchus ever reported. Because of its intraluminal growth, typical bronchial carcinoid may also be treated bronchoscopically with curative intent (17,18).

Recurrent GCMs were was probably due to inadequate assessment of peribronchial tumor extension before treatment.

The morbidity of bronchoscopic treatment is low, so bronchoscopic therapy should be attempted first in patients with intraluminal lesions. This is in accordance with several other reports published previously, justifying a more conservative approach toward tracheobronchial GCM, even if localizations are multiple (4,6,8).

In summary, bronchoscopic treatment of GCM may be attempted as the first approach for intraluminal lesions not larger than 1 cm without peribronchial growth and in the absence of irreversible damage of lung parenchyma.

### REFERENCES

1. McLain WC, Olsen GN, Wooldridge D, et al. Endotracheal granular cell myoblastoma. A failure of laser therapy. Chest 1984;86:136–137.
2. O'Connell DJ, McMahon H, De Meester TR. Multicentric tracheobronchial and oesophageal granular cell myoblastoma. Thorax 1979;33:596–602.
3. Redjaee B, Rohatgi PK, Herman MA. Multicentric endobronchial granular cell myoblastoma. Chest 1990;98:945–948.
4. Valenstein SL, Thurer RJ. Granular cell myoblastoma of the bronchus. Case report and literature review. J Thorac Cardiovasc Surg 1978;76:465–468.
5. Sobel HJ, Marquet E, Avrin E, et al. Granular cell myoblastoma. Am J Pathol 1971;65:59–71.
6. Daniel TM, Smith RH, Faunce HF, et al. Transbronchoscopic versus surgical resection of tracheobronchial granular cell myoblastomas. Suggested approach based on follow-up of all treated cases. J Thorac Cardiovasc Surg 1980;80:898–903.
7. Lack EE, Worsham GM, Callihan MD, et al. Granular cell tumor: a clinico-pathologic study of 110 patients. J Surg Oncol 1980;13:301–316.
8. Schwartzberg DG, Al Bazzaz FJ, Cassel J, et al. Multiple granular cell tumors of the bronchi: treatment with laser. Am Rev Respir Dis 1979;120:193–196.
9. Aberle DR, Brown K, Young DA, et al. Imaging techniques in the evaluation of tracheobronchial neoplasms. Chest 1991;99:211–215.
10. Sutedja G, Golding RP, Postmus PE. High resolution computed tomography in patients referred for intraluminal bronchoscopic therapy with curative intent. Eur Respir J, in press.
11. Coleman BG, Arger PH, Stephenson LW. CT features of endobronchial granular cell myoblastoma. J Comput Assist Tomogr 1984;8:998–1000.
12. Edell ES, Cortese DA. Photodynamic therapy in the management of early superficial squamous cell carcinoma as an alternative to surgical resection. Chest 1992;102:1319–1322.
13. Sutedja G, Postmus PE. Review article. Bronchoscopic treatment of lung tumors. Lung Cancer 1994;11:1–17.
14. Epstein LJ, Mohsenifar Z. Use of Nd:YAG laser in endobronchial granular cell myoblastoma. Chest 1993;104:958–960.
15. Cadotte M. Malignant granular cell myoblastoma. Cancer 1974;33:1417–1422.
16. Sutedja G, Schreurs AJ, Vanderschueren RG, et al. Bronchoscopic therapy in patients with intraluminal typical carcinoid. Chest 1995;107:556–558.
17. Sutedja G, Schramel F, Postmus PE. Bronchoscopic treatment of intraluminal typical carcinoid. Eur Respir J 1995;8(Suppl 19):308S.
18. Sutedja G, Schramel F, Postmus PE. Bronchoscopic electrosurgery with curative intent for intraluminal and roentgenologically occult lung tumours. Eur Respir J 1995;8(Suppl 19):307S.

